# The prevalence and associated factors of dry eye disease amongst people diagnosed with diabetes mellitus in Bronkhorstspruit

**DOI:** 10.3389/fcdhc.2026.1760534

**Published:** 2026-03-30

**Authors:** Tlou K Mefane, Cairo B. Ntimana, Reneilwe G. Mashaba, Eric Mailmela

**Affiliations:** 1 Department of Public Health, Polokwane, South Africa; 2 Dikage Mababolo Mothiba (DIMAMO) Population Health Research Centre, University of Limpopo, Polokwane, South Africa; 3 Department of Public Health, Walter Sisulu University, Eastern Cape, South Africa

**Keywords:** diabetes, dry eye, education, female’, ocular surface disease index

## Abstract

**Background:**

Dry eye disease (DED) is a common ocular surface disorder with a significant impact on quality of life. Its prevalence is higher among individuals with diabetes mellitus (DM), yet limited evidence exists in African settings, particularly in rural communities. Hence, the present study aimed to determine the prevalence and associated factors of DED among individuals living with diabetes in Bronkhorstspruit, South Africa.

**Methods:**

A cross-sectional study was conducted between November 2024 and February 2025 among 236 diabetic patients selected through simple random sampling from a public hospital and a private clinic. Standardized diagnostic tests (tear break-up time [TBUT], Schirmer test) and the Ocular Surface Disease Index (OSDI) questionnaire were used to diagnose and grade DED. Socio-demographic and ocular history data were collected. Logistic regression was performed to identify determinants of DED, with results reported as odds ratios (ORs) at 95% confidence intervals (CIs).

**Results:**

Of the 163 participants analyzed, the prevalence of DED was 81.6%. Prevalence increased with age, with the ≥65 years group most affected (Males: 50.0%; Females: 38.4%). Participants aged ≥45 years were 4.5–9.1 times more likely to develop DED compared to those aged ≤44 years. Females reported higher rates of dryness (24.2%), tearing (10.1%), and itching (6.1%), while males reported more blurry vision (68.8%) and pain (14.1%). Lower educational attainment was associated with a 2.1-fold increased risk of severe DED. Marital status and ocular comorbidities were not significantly associated with DED.

**Conclusion:**

The prevalence of DED among individuals living with diabetes in Bronkhorstspruit was 81.6%. An increase with age, female sex, and lower educational attainment were identified as factors significantly associated with DED, whereas marital status was not associated with DED. The high prevalence reported underscores the urgent need for context-specific interventions, including community-level awareness, early screening, and tailored management strategies, to mitigate the ocular complications of diabetes. Future studies should explore environmental exposures as additional risk factors.

## 1 Introduction

Dry eye disease is one of the most prevalent ocular surface disorders, with a global prevalence of 11.59% and even higher prevalences in Africa (42.0%) and Asia (20.1%) (1). In America, the prevalence of DED varies considerably, ranging from 4.3% to 21.6% (2–5). The prevalence of DED is further reported to be higher in children (16.6%), thus making it a public health concern (5). A study in a rural Nigerian community reported a prevalence of 27.4% (6). In Sohag City, southern Egypt, the prevalence was 22.8% (7). Although such figures underscore the impact of DED in developing regions, a critical gap remains in epidemiological data, particularly in African countries. Studies have emphasized that DED constitutes a significant public health issue in Africa (8–10). Nonetheless, the available data are fragmented and insufficient (9).

Despite the high prevalence, the impact of DED on quality of life is often underestimated (11). DED is typically classified into two major types: aqueous-deficient dry eye, characterized by reduced tear production, and evaporative dry eye, which results from excessive evaporation of the tear film (12–14). Both forms are marked by increased tear film osmolarity and reduced TBUT, indicating compromised tear film quality and stability (15, 16). An epidemiological study found an unusually high DED prevalence in a private healthcare setting, further highlighting the disparity and the urgent need for broader, community-based investigations (17).

The lack of comprehensive and localized data on DED, especially among individuals with comorbid conditions such as diabetes mellitus, is a problem, especially in rural African communities. Diabetes mellitus (DM) has been identified as a risk factor for numerous ocular complications, through its impact on the structure of the cornea and adverse effects on the ocular surface (18). Chronic hyperglycemia, impairment of insulin secretion, and corneal nerve damage are proposed mechanisms for the alterations in the tear film and ocular surface of patients (18). The present study aimed to determine the prevalence and identify factors associated with DED among individuals diagnosed with diabetes mellitus in Bronkhorstspruit. Understanding the prevalence and determinants of DED in Bronkhorstspruit will help bridge the knowledge gap and inform more effective clinical and public health responses.

## 2 Materials and methods

### 2.1 Study design

The present study employed a cross-sectional design and used simple random sampling to select participants from Focus Optometrists (a private clinic) and Bronkhorstspruit Hospital (a public hospital).

### 2.2 Sampling

A sampling frame was created by compiling all diabetic patients recorded in the two facility registers during the study period. Clinic registers, appointment logs, and electronic records were merged to produce a complete list of 436 diabetic patients. After removing duplicate entries and individuals who did not meet basic eligibility criteria (non-diabetic or below 18 years), a final sampling frame of 236 eligible patients was obtained. Simple random sampling was then performed using a computerized random-number generator in Microsoft Excel. Each patient was assigned a unique study ID, random values were generated using the RAND() function, and the list was sorted in ascending order. The first 236 individuals on the randomized list were selected and invited to participate in the study.

### 2.3 Eligibility criteria and participant exclusions

**Figure 1:**
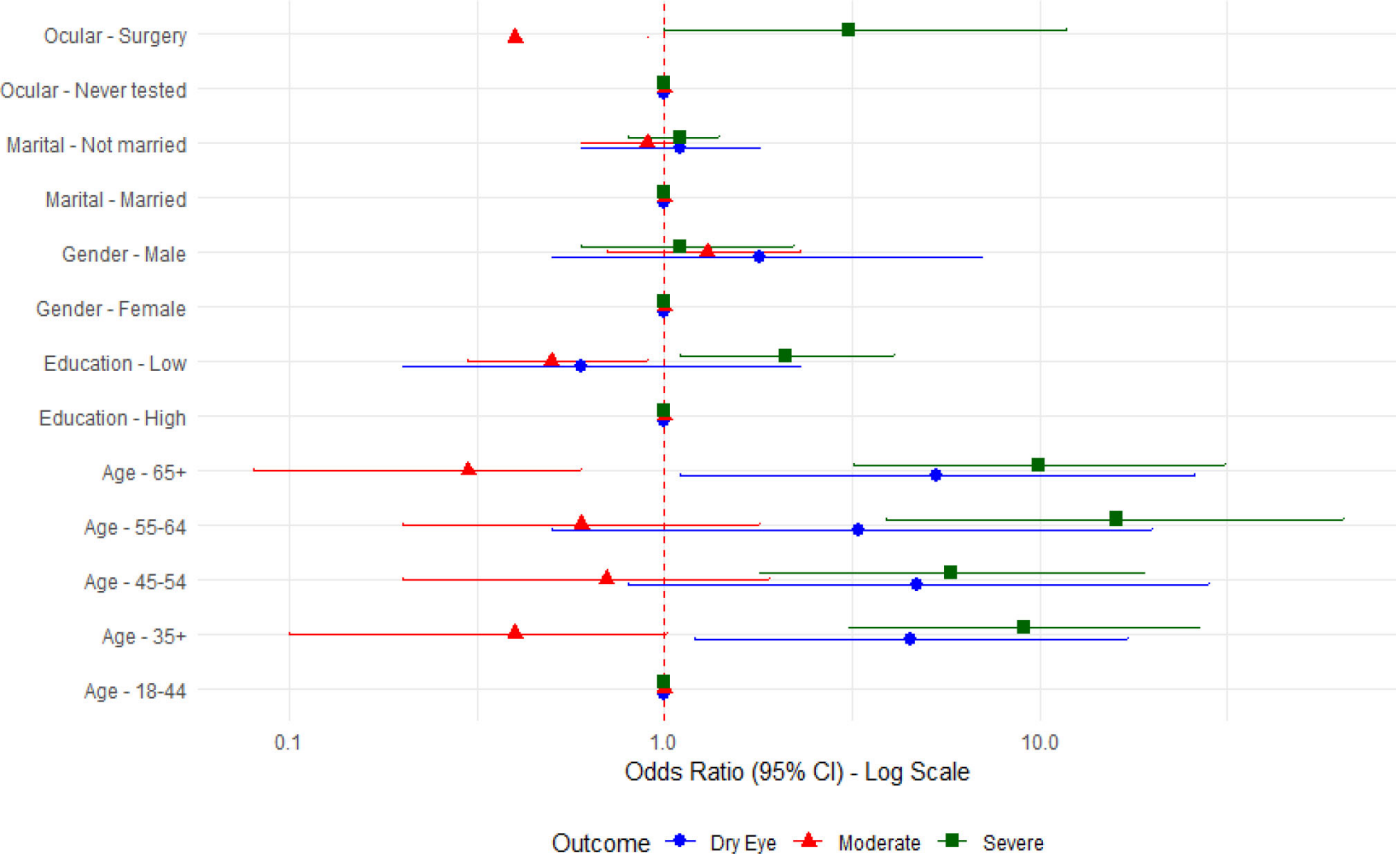
Selection criteria.

Eligible participants included adults (≥18 years) diagnosed with diabetes mellitus who were able to complete dry-eye assessments. Exclusion criteria included a history of Sjögren’s syndrome, rheumatoid arthritis, recent ocular infection or surgery, pregnancy, contact-lens use, or current use of topical/systemic medications known to affect tear-film stability. Of the 236 randomly selected eligible participants, 73 (30.9%) were excluded during clinical assessment. Exclusions were protocol-driven and occurred due to incomplete objective tear-film measurements (missing TBUT and/or Schirmer results), incomplete OSDI questionnaires, or identification of predefined exclusion criteria (e.g., undisclosed contact lens use, recent ocular surgery, or medications affecting tear-film stability). Because dry eye diagnosis required complete clinical and questionnaire data, only participants with full datasets were included in the final analysis (n = 163). A review of available demographic register data (age and sex) showed no meaningful differences between included and excluded participants, reducing the likelihood of systematic selection bias (**Figure 1**).

### 2.4 Data collection

Data was collected through quality assessment of the anterior chamber, TBUT, and Schirmer test. The OSDI questionnaire is a 12-item instrument developed in 1997. Collected data downloaded from browsers and checked for completeness and consistency before merging with Microsoft Excel. A data collection tool questionnaire from the OSDI questionnaire was designed after a pilot data collection tool was created to assess subjective dry eye symptoms and the effects of DED on vision-related activities. A data collection tool questionnaire from the OSDI questionnaire was designed after a pilot data collection tool was created to assess subjective dry eye symptoms and the effects of DED on vision-related activities. The Ocular Surface Disease Index (OSDI) is a validated 12-item instrument comprising three subscales: (1) ocular symptoms, (2) vision-related function, and (3) environmental triggers. Each item is scored on a 5-point Likert scale (0–4), and the total score is calculated using the standard OSDI formula, with higher scores indicating greater symptom severity.

### 2.5 Determination of DED

DED was assessed using the Ocular Surface Disease Index (OSDI), TBUT, and Schirmer I test without anesthesia (12). The OSDI was scored according to established thresholds: <13 (normal), 13–22 (mild), 23–32 (moderate), and ≥33 (severe). A TBUT of ≤10 seconds and a Schirmer I reading of ≤10 mm/5 minutes were used as diagnostic indicators of DED, with values ≤5 representing severe tear-film instability or aqueous deficiency. DED was operationally defined as the presence of clinically significant symptoms (OSDI ≥13) together with at least one objective tear film abnormality (TBUT ≤10 seconds or Schirmer I ≤10 mm/5 minutes). Due to resource limitations, tear osmolarity testing and ocular surface staining, as recommended by the Tear Film & Ocular Surface Society Dry Eye Workshop II (TFOS DEWS II), were not performed.

### 2.6 Statistical analysis

Data analysis was conducted using STATA version 15 for Windows (StataCorp LP, College Station, TX, USA). Descriptive statistics were used to summarize participant characteristics. Categorical variables were presented as frequencies and percentages with corresponding 95% confidence intervals (CIs). Univariable logistic regression was first performed to identify candidate variables associated with clinically significant dry eye disease (p < 0.20). Variables meeting this threshold, together with variables considered clinically relevant based on prior literature (age, sex, educational status), were entered simultaneously into a multivariable logistic regression model to adjust for potential confounding. No stepwise selection procedures were applied. Multicollinearity was assessed using variance inflation factors (VIF), with all VIF values < 2.5, indicating no significant collinearity. Moderate and severe dry eye categories were combined to define clinically significant DED to ensure adequate statistical power and model stability, given the distribution of severity categories and small cell counts in subgroup analyses. This approach aligns with epidemiological practice, where moderate-to-severe disease is considered clinically meaningful. Statistical significance was set at p < 0.05.

## 3 Results

The present study analysed data from 163 participants, of whom the majority were female (60.8%), with a mean age of 59.0 ± 13.5 years. Most participants were married (45.4%) and employed (68.7%), although these differences were not statistically significant, with *p*-values of 0.178 and 0.295, respectively (**Table 1**).

**Table 1:** Participants’ characteristics.

Socio-demographic profile	Total	Males (n=64)	Females (n=99)	P-values
	n (%)	n (%)	n (%)	
Age group in years				
18-34	2 (1.2)	1 (1.6)	1 (1.0)	0.405
35-44	19 (11.7)	6 (9.4)	13 (13.1)	
45-54	42 (25.8)	12 (18.8)	30 (30.3)	
55-64	30 (18.4)	13 (20.3)	17 (17.2)	
≥ 65	70 (42.9)	32 (50.0)	38 (38.4)	
Mean age	59.0 ± 13.5	61.8 ± 12.5	57.2 ± 14.0	0.405
Marital status				
Single	32 (19.6)	10 (15.6)	22 (22.2)	0.178
Married	74 (45.4)	35 (54.7)	39 (39.4)	
Divorced	33 (20.3)	9 (14.1)	24 (24.2)	
Widowed	24 (14.7)	10 (15.6)	14 (14.1)	
Employment status				
Unemployed	51 (31.3)	17 (26.6)	34 (34.3)	0.295
Employed	112 (68.7)	47 (73.4)	65 (65.7)	

In the total population, 60.7% had a higher proportion of blurry vision, with males having a significantly higher prevalence of 68.8%, compared to females at 55.6%. Conversely, females experienced dryness at a higher rate, with a proportion of 24.2% compared to 9.4% in males. Both tearing and pain affected 7.4% of the total population, with males experiencing more pain (14.1%) than females (3.1%). Females reported higher instances of tearing at 10.1% than males at 3.1%. Additionally, 3.7% of the total population reported itching, with a higher proportion of 6.1% among females than males. In the subgroup without ocular symptoms, which made up 2.5% of the population, males accounted for 4.7%, compared to females 1%. In the study population, 39.3% had never been tested, with a higher proportion among females (50.0%) compared to males (21.9%). Conversely, 38.7% of participants who wore spectacles had DED, which was more prevalent among males (46.9%) than females (33.3%). The proportion of participants who had undergone eye surgery was also higher in males (15.9%) than in females. Among those diagnosed with cataracts (4.9% of the population), a higher proportion were males (9.4%) compared to females (2%). Glaucoma was present in 1.2% of the population, with a slightly higher prevalence among males (1.2%) than females (1.0%) (**Table 2**).

**Table 2:** The ocular symptoms and ocular status of participants stratified by gender.

	Total	Males (n=64)	Females (n=99)	P-values
	n (%)	n (%)	n (%)	
Ocular symptoms				
None	4 (2.5)	3 (4.7)	1 (1.0)	0.001
Dry	30 (18.4)	6 (9.4)	24 (24.2)	
Tearing	12 (7.4)	2 (3.1)	10 (10.1)	
Pain	12 (7.4)	9 (14.1)	3 (3.1)	
Blurry	99 (60.7)	44 (68.8)	55 (55.6)	
Itching	6 (3.7)	0 (0.0)	6 (6.1)	
Ocular status				
Never tested	64 (39.3)	14 (21.9)	50 (50.5)	0.003
Surgery	26 (15.9)	13 (20.3)	13 (13.1)	
Glaucoma	2 (1.2)	1 (1.6)	1 (1.0)	
Cataract	8 (4.9)	6 (9.4)	2 (2.0)	
Wearing spectacles	63 (38.7)	30 (46.9)	33 (33.3)	

**Table 3** presents the overall prevalence of clinically diagnosed dry eye DED stratified by age group, gender, marital status, and ocular status. The prevalence of DED was found to increase with age across all groups. Those aged 65 and above had the highest proportion of DED (males = 50% and females 38.4%). Married and widowed individuals had a higher proportion of DED in older age groups, with widowed participants peaking at 75.0%. Those who had never tested their eyes or had undergone eye surgery also showed increasing DED rates with age. Notably, participants with cataracts or glaucoma had particularly high DED prevalence, especially in older age groups. Wearing spectacles was also associated with a higher proportion of DED in older adults.

**Table 3:** Overall prevalence of clinically diagnosed dry eye DED stratified by age group, gender, marital status, and by ocular status.

Variables	Age in years				
	18-34	35-44	45-54	55-64	≥ 65
	% (95% CI)	% (95% CI)	% (95%CI)	% (95% CI)	% (95% CI)
Gender					
Males	1.5 (0.2 – 10.6)	9.4 (4.2 – 19.6)	18.8 (10.8 – 30.4)	20.3 (12.0 – 32.2)	50.0 (37.8 – 62.2)
Females	1.0 (0.1 – 6.9)	13.1 (7.7 – 21.4)	30.3 (21.9 – 40.2)	17.2 (10.9 – 26.0)	38.4 (29.2 – 48.4)
Marital status					
Single	6.3 (1.5 – 22.8)	28.1 (14.9 – 46.6)	53.1 (35.5 – 70.0)	3.1 (0.4 – 20.4)	9.4 (2.9 – 26.3)
Married	–	12.2 (6.3 – 21.9)	21.6 (13.6 – 32.6)	24.3 (15.8 – 35.5)	41.9 (31.1 – 53.6)
Widowed	–	4.2 (0.5 – 26.5)	4.2 (0.5 – 26.5)	16.7 (6.1 – 38.3)	75.0 (53.1 – 88.8)
Divorced	–	–	24.2 (12.3 – 42.3)	21.2 (10.2 – 39.1)	54.5 (37.1 – 70.9)
Ocular status					
Never tested	1.6 (0.2 – 10.6)	23.4 (14.5 – 35.6)	35.9 (24.9 – 48.6)	15.6 (8.5 – 26.9)	23.4 (14.5 – 35.6)
Surgery	–	3.8 (0.5 – 24.6)	7.7 (1.8 – 27.5)	11.5 (3.6 – 31.6)	76.9 (56.1 – 89.7)
Glaucoma	–	–	50 (1.57e – 1)	50 (1.57e – 1)	–
Cataract	–	–	12.5 (1.1 – 64.2)	12.5 (1.1 – 64.2)	75.0 (30.3 – 95.4
Wearing spectacles	1.6 (0.2 – 10.8)	4.8 (1.5 – 14.0)	23.8 (14.7 – 36.1)	23.8 (14.7 – 36.1)	46.0 (33.9 – 58.6)

**Table 4** indicates that the proportion of severe DED was 60.6% among females using the TBUT test and 46.5% using the Schirmer test. Among males, the proportion of severe DED was 64.1% based on TBUT and 48.4% based on the Schirmer test. Moderate DED among males was 29.7% according to TBUT and 40.6% according to the Schirmer test, while the proportion classified as normal was 6.3% (TBUT) and 10.9% (Schirmer). These findings indicate a high proportion of severe DED in both males and females based on objective tear-film assessments. Among females, moderate DED was reported at 29.3% using TBUT and 33.3% using the Schirmer test. Overall, severe DED was observed in both sexes across both diagnostic tests, with similar distribution patterns.

**Table 4:** The difference between the quality and the quantity of tears amongst the participants using the TBUT and the Schirmer test.

	TBUT n (%)	Schirmer n (%)	P-value
Females			
Normal	10 (10.1)	20 (20.2)	<0.001
Moderate	29 (29.3)	33 (33.3)	
Severe	60 (60.6)	46 (46.5)	
Males			
Normal	4 (6.3)	7 (10.9)	<0.001
Moderate	19 (29.7)	26 (40.6)	
Severe	41 (64.1)	31 (48.4)	

**Table 5** shows the factors associated with clinically significant dry eye disease (moderate or severe). Increasing age was independently associated with higher odds of clinically significant DED. Compared to participants aged 18–44 years, those aged 45–54, 55–64, and ≥65 years had progressively higher odds, with the strongest association observed in the ≥65 years group (AOR = 9.9; 95% CI: 3.2–31.3; p < 0.001). Lower educational attainment was also significantly associated with increased odds of clinically significant DED (AOR = 2.1; 95% CI: 1.1–4.1; p < 0.05). Gender, marital status, ocular surgery history, glaucoma, and spectacle use were not significantly associated after multivariable adjustment.

**Table 5:** Determinants of Clinically Significant DED.

Variable	Crude OR (95% CI)	Adjusted OR (95% CI)	P-value
Age (years)			
18–44	Reference	Reference	—
45–54	4.7 (0.8–28.2)	4.5 (1.2–17.1)	0.026*
55–64	3.3 (0.5–19.9)	5.8 (1.8–18.9)	0.003**
≥65	5.3 (1.1–25.7)	9.9 (3.2–31.3)	<0.001***
Gender			
Female	Reference	Reference	—
Male	1.8 (0.5–7.0)	1.3 (0.7–2.3)	0.412
Educational status			
High	Reference	Reference	—
Low	1.4 (0.8–2.6)	2.1 (1.1–4.1)	0.021*
Marital status			
Married	Reference	Reference	—
Not married	1.1 (0.6–1.8)	1.0 (0.7–1.5)	0.834
Ocular surgery history			
No	Reference	Reference	—
Yes	1.6 (0.8–3.2)	1.4 (0.7–2.8)	0.298
Glaucoma			
No	Reference	Reference	—
Yes	2.4 (0.6–9.8)	2.1 (0.5–8.7)	0.309
Wearing spectacles			
No	Reference	Reference	—
Yes	1.2 (0.7–2.1)	1.1 (0.6–2.0)	0.721

* p < 0.05; ** p < 0.01; *** p < 0.001.

## 4 Discussion

The present study aimed to determine the prevalence and factors associated with DED among people diagnosed with diabetes in Bronkhorstspruit. The prevalence of DED among individuals with diabetes was 81.6%. This high prevalence reflects a substantial burden of ocular surface abnormalities in this clinic-attending diabetic population. Previous literature has reported that DED is more common in individuals with diabetes compared to non-diabetic populations (19), likely due to chronic hyperglycaemia and its microvascular and neuropathic effects. In addition to chronic hyperglycaemia, the high DED prevalence in diabetes has been associated with elevated glycosylated haemoglobin levels, oxidative stress, and longer duration of diabetes (19, 20). Recent evidence has also demonstrated alterations in the ocular surface microbiome among diabetic patients with dry eye (21), suggesting a multifactorial pathophysiological process. The prevalence observed in the present study is comparable to findings reported in South Africa (82.3%) (22) and substantially higher than the estimated global prevalence of 11.59% (23), highlighting the need for context-specific screening strategies in diabetic populations. This difference should be interpreted cautiously, as global pooled estimates predominantly reflect general population samples, whereas the present study focused exclusively on clinic-attending individuals with diabetes. The older age distribution of this cohort and the clinical setting may have contributed to higher case detection compared to community-based studies.

Age was significantly associated with DED in this study, with participants aged 45 years or older showing higher odds of clinically significant dry eye compared to younger participants. Aging is associated with structural and functional changes in the lacrimal gland, increased inflammatory activity, and reduced tear-film stability (24). Similar associations between advancing age and DED have been reported in other populations (25, 26). These findings support the need for routine ocular surface assessment in older adults living with diabetes.

An important observation in the present study was the apparent discordance between subjective symptoms and objective clinical findings by sex. Females reported higher frequencies of dryness, tearing, and itching, whereas males demonstrated a higher proportion of severe tear-film abnormalities based on TBUT and Schirmer testing. This divergence between symptom reporting and objective measurements has been described in dry eye research and underscores the complexity of DED assessment. Several mechanisms may explain this discrepancy. Women may be more likely to report ocular discomfort due to greater health-seeking behaviour or hormonal influences affecting tear-film stability, particularly related to estrogen fluctuations. Conversely, males with diabetes may exhibit more pronounced objective tear-film instability, potentially linked to diabetic neuropathy, which can reduce corneal sensitivity and alter symptom perception despite measurable ocular surface damage. These findings highlight the importance of integrating both subjective symptom questionnaires and objective tear-film assessments when evaluating DED, particularly in diabetic populations where neuropathic changes may influence symptom reporting.

Educational status showed a mixed association pattern. Participants with lower education had lower odds of moderate DED but higher odds of severe DED. This may reflect delayed healthcare-seeking behaviour or reduced awareness of early symptoms among individuals with lower educational attainment (27). However, this interpretation should be approached cautiously, given the study’s cross-sectional design. Marital status was not significantly associated with DED in the multivariable analysis, and subgroup estimates demonstrated wide confidence intervals, suggesting statistical instability. Given the small numbers within certain marital subcategories across age groups, percentage estimates were highly variable and should be interpreted cautiously. Therefore, marital status was not considered a meaningful predictor in this population.

Glaucoma, cataract, and spectacle use were not significantly associated with DED in the multivariable analysis. Although some studies have suggested associations between ocular comorbidities and dry eye (8, 28). These relationships were not observed in the present population after adjustment for confounding factors. Due to the cross-sectional nature of the study, causal relationships cannot be inferred, and the findings should be interpreted strictly as associations rather than determinants. Longitudinal studies are warranted to better understand the temporal relationship between diabetes-related factors and the progression of DED.

Although environmental factors such as dust exposure, wind, ultraviolet radiation, and air pollution have been reported in the literature as potential contributors to dry eye symptoms (8, 28), these variables were not assessed in the present study. Therefore, their role in the observed high prevalence cannot be determined from our data. In the context of Bronkhorstspruit and similar semi-rural environments, such exposures may plausibly interact with diabetes-related ocular surface vulnerability; however, this interpretation should be regarded as hypothesis-generating rather than explanatory. Future studies should incorporate direct measurement of environmental and occupational exposures to better understand their contribution to DED in diabetic populations.

### Limitations and strengths of the study

We employed the Schirmer and TBUT tests (recognized as reliable and practical indicators of DED). Similarly, variables such as glycaemic control and microvascular complications were not consistently available from participant records. Thus, they were excluded. Due to the cross-sectional design, causal relationships cannot be inferred, and the findings should be interpreted as associations rather than determinants. Environmental and lifestyle factors that could influence DED were not assessed. The study had potential recall bias as it adopted a self-reported approach to symptoms. The current international standard for dry eye classification (TFOS DEWS II diagnostic framework) was not used due to limited resources. Although OSDI, TBUT, and Schirmer I tests are widely used in epidemiological studies, the absence of tear osmolarity and ocular surface staining may have increased the sensitivity of case detection and limits strict comparability with studies applying the full TFOS DEWS II diagnostic algorithm. Approximately 30.9% of randomly selected participants were excluded due to incomplete clinical or questionnaire data. Although demographic characteristics available from clinic registers did not indicate major differences between included and excluded participants, some degree of selection bias cannot be entirely excluded. Therefore, findings should be interpreted as generalizable to facility-attending diabetic patients rather than the broader diabetic community. Nevertheless, the study has provided insightful findings since it is the first to investigate DED and its determinants in rural South Africa. Multivariate logistic regression strengthened the analysis by adjusting for potential confounders.

## 5 Conclusions

The prevalence of DED among individuals living with diabetes in Bronkhorstspruit was high (81.6%), indicating a substantial burden in this clinic-based population. Increasing age and lower educational status were independently associated with higher odds of DED, whereas marital status was not significantly associated after multivariable adjustment. These findings support the integration of routine ocular surface assessment into diabetic care, particularly for older adults. Although environmental exposures were not assessed in this study, future research should investigate localized and contextual risk factors to better understand potential contributors to disease burden. Further longitudinal studies incorporating comprehensive diagnostic protocols, such as those recommended by the Tear Film & Ocular Surface Society Dry Eye Workshop II (TFOS DEWS II), together with detailed diabetic profiling, are warranted to strengthen causal inference and improve clinical management strategies.

## Data Availability

The raw data supporting the conclusions of this article will be made available by the authors, without undue reservation.
